# Definitive chemoradiotherapy in patients with squamous cell cancers of the head and neck - results from an unselected cohort of the clinical cooperation group “Personalized Radiotherapy in Head and Neck Cancer”

**DOI:** 10.1186/s13014-019-1452-4

**Published:** 2020-01-06

**Authors:** Lars Schüttrumpf, Sebastian Marschner, Katrin Scheu, Julia Hess, Sibylle Rietzler, Axel Walch, Philipp Baumeister, Thomas Kirchner, Ute Ganswindt, Horst Zitzelsberger, Claus Belka, Cornelius Maihoefer

**Affiliations:** 1 Clinical Cooperation Group ‘Personalized Radiotherapy in Head and Neck Cancer’, Helmholtz Zentrum München, German Research Center for Environmental Health GmbH, 85764 Neuherberg, Germany; 2 Department of Radiation Oncology, University Hospital, LMU Munich, Marchioninistr 15, 81377 Munich, Germany; 3 Research Unit Radiation Cytogenetics, Helmholtz Zentrum München, German Research Center for Environmental Health GmbH, 85764 Neuherberg, Germany; 4 Institute of Pathology, Faculty of Medicine, LMU Munich, Marchioninistr 27, 81377 Munich, Germany; 5 Research Unit Analytical Pathology, Helmholtz Zentrum München, German Research Center for Environmental Health GmbH, 85764 Neuherberg, Germany; 6 Department of Otorhinolaryngology, Head and Neck Surgery, University Hospital, LMU Munich, Marchioninistr 15, 81377 Munich, Germany; 7 Department of Radiation Oncology, University of Innsbruck, Anichstraße 35, 6020 Innsbruck, Austria

**Keywords:** Head and neck cancer, Defintive, Primary, Chemoradiation, Radiotherapy, HNSCC, HPV

## Abstract

**Background:**

Definitive chemoradiotherapy (dCRT) is a standard treatment for patients with locally advanced head and neck cancer. There is a clinical need for a stratification of this prognostically heterogeneous group of tumors in order to optimize treatment of individual patients. We retrospectively reviewed all patients with head and neck squamous cell carcinoma (HNSCC) of the oral cavity, oropharynx, hypopharynx, or larynx, treated with dCRT from 09/2008 until 03/2016 at the Department of Radiation Oncology, LMU Munich. Here we report the clinical results of the cohort which represent the basis for biomarker discovery and molecular genetic research within the framework of a clinical cooperation group.

**Methods:**

Patient data were collected and analyzed for outcome and treatment failures with regard to previously described and established risk factors.

**Results:**

We identified 184 patients with a median follow-up of 65 months and a median age of 64 years. Patients received dCRT with a median dose of 70 Gy and simultaneous chemotherapy in 90.2% of cases, mostly mitomycin C / 5-FU in concordance with the ARO 95–06 trial. The actuarial 3-year overall survival (OS), local, locoregional and distant failure rates were 42.7, 29.8, 34.0 and 23.4%, respectively. Human papillomavirus-associated oropharynx cancer (HPVOPC) and smaller gross tumor volume were associated with significantly improved locoregional tumor control rate, disease-free survival (DFS) and OS in multivariate analysis. Additionally, lower hemoglobin levels were significantly associated with impaired DFS und OS in univariate analysis. The extent of lymph node involvement was associated with distant failure, DFS and OS. Moreover, 92 patients (50%) of our cohort have been treated in concordance with the ARO 95–06 study, corroborating the results of this study.

**Conclusion:**

Our cohort is a large unselected monocentric cohort of HNSCC patients treated with dCRT. Tumor control rates and survival rates compare favorably with the results of previously published reports. The clinical data, together with the available tumor samples from biopsies, will allow translational research based on molecular genetic analyses.

## Introduction

Head and Neck Cancer is the seventh most common type of cancer in the world. In Europe, Head and Neck Cancer accounts for an estimated 145,000 new cases every year [1]. Definitive chemoradiotherapy (dCRT) is a standard-of-care treatment for locoregional advanced head and neck squamous cell cancer (HNSCC). A simultaneous treatment by chemotherapy and radiotherapy turned out to be the most effective option and leads to an improvement of the overall survival (OS) of around 5% [2]. The prognosis of the patients varies depending on risk factors such as tumor localization, the size of the primary tumor, the extent of the lymph node involvement and tumor hypoxia [3]. Moreover, in recent years, the identification of human papillomavirus-associated tumorigenesis in oropharyngeal cancer (HPVOPC) has proven to be one of the most important prognostic factors [4]. Avoidable major risk factors include smoking and alcohol abuse. Smokers are ten times more likely to develop HNSCC than non-smokers [5]. Depending on the tumor biology and the risk factors mentioned, HNSCC vary widely in response to therapy and prognosis for the patient [6–9].

Further research is still crucial to establish biomarkers enabling a tailored, risk-adapted use of the available treatment modalities. To achieve this goal, a solid database of a HNSCC cohort is necessary for our translational research in the framework of the multidisciplinary translational Clinical Cooperation Group ‘Personalized Radiotherapy in Head and Neck Cancer’.

## Material & Methods

We retrospectively analyzed patients with squamous cell carcinoma of the oral cavity, oropharynx, hypopharynx and larynx who have been treated with dCRT in our clinic (Department of Radiation Oncology, Ludwig-Maximilians-University Munich – LMU) between 09/2008 until 03/2016.

Until 2013 CT-based three-dimensional planning was used to generate radiation plans with a sequential boost for therapeutic planning target volume (PTV) prescribing a median dose of 50 Gy for prophylactic lymph node level, 60 Gy for involved lymph node level and 70 Gy for therapeutic target volume (primary tumor and suspicious lymph nodes). Patients were treated 5 days a week with 2 Gy per fraction. Since 2013 patients were treated by a simultaneous integrated boost (SIB) using IMRT / VMAT [10]. A median dose of 70 Gy (66 /69.96 / 70 / 70.4) was prescribed to the therapeutic target volume in 32–35 fractions of 2 to 2.2 Gy. A median dose of 50.4 to 54.45 Gy was prescribed to the prophylactic lymph node levels.

Most patients received additional chemotherapy. Department standard was Mitomycin C / 5-FU in concordance with ARO 95–06 (Mitomycin C was administered as a single intravenous bolus injection of 10 mg/m^2^ on days 5 and 36, 5-FU was administered as a continuous infusion for 120 h at 600 mg/m^2^/d on days 1 to 5).

This standard was changed to CDDP weekly in 2013 (40 mg/^2^ on day 2, 8, 15, 22, 29, 36, 43). Other chemotherapeutic regimens (such as Carboplatin, Mitomycin C mono or Cetuximab) were used if a patient was not suitable for department standard. Due to comorbidities and reduced general condition, some patients were treated with radiotherapy alone.

The clinic’s radiation therapy management system (Mosaiq® - Elekta, Sweden) and patient files recorded in a dedicated Microsoft Access Relational Database were used to collect patient data.

Tumor stage was assessed using the UICC 2010 TNM classification, if not stated otherwise. Immunohistochemical (IHC) p16INK4a staining results from our local pathology was used as a surrogate marker for HPV-infection, if available (75 patients). Additionally, 81 HNSCC patients were analyzed for HPV p16 within the framework of the KKG. IHC p16INK4a staining was performed using the CINtec TM Histology Kit (Roche mtm laboratories AG, Germany) on a Ventana Benchmark LT automated immunostainer (Ventana Medical Systems, Tucson AZ, USA) according to the protocol. Strong and diffuse nuclear and cytoplasmic staining in > 70% of tumor cells were considered as p16-positive.

Follow-up data has been collected in the joint survivorship clinic of the Otorhinolaryngological and the Radiation Oncology Department of the LMU, but also from follow-up visits in our clinic or by phone interviews.

Follow-up has been calculated from the last day of radiation therapy with the inverse Kaplan-Meier method. All other endpoints such as survival or time to recurrence have been calculated from the first day of the radiation treatment. The events of the survival endpoints were defined as following: OS – death, DFS – death or any recurrence, DSS – only death related to recurring HNSCC. *P*-values were determined using log-rank testing for comparison between groups. Univariate and multivariate analyses were conducted using Cox proportional hazard regression models. If more than one factor was significant in univariate Cox regression analysis, multivariate Cox regression analysis was used for determining the influence of multiple covariates. Statistical analyses were performed with SPSS V25 (IBM, Chicago, IL). *P*-values of < 0.05 were considered statistically significant. Median estimates and Hazard ratios (HR) with 95% confidence intervals (CI) were determined. Ethics approval for collecting patient-derived data and investigating tumor samples by molecular genetic approaches were granted by the local ethics committee of the LMU Munich (No. 448–13, 459–13, 17–116).

## Results

### Patient and treatment characteristics

A total of 184 patients with HNSCC of the oral cavity, oropharynx, hypopharynx and larynx were treated with dCRT at the Department of Radiation Oncology of the LMU between 09/2008 until 03/2016. Patient, tumor and treatment characteristics are shown in Table 1. The median age was 64 years (range 23–89 years) at time of diagnosis. The median follow-up was 65.0 months. 97% of patients completed radiation therapy and received at least 66 Gy to primary tumor. Median cumulative dose was 70 Gy. Nine patients (4.9%) were treated with hyperfractionated accelerated radiotherapy. 90.2% of patients (*n* = 166) received concurrent systemic therapy.

**Table 1:** Patient and treatment characteristics for all patients (left panel), ARO-analogue subgroup (middle panel) and HPVOPC (right panel)

Factors	All patients		ARO-analogue		HPVOPC	
	*n* = 184		*n* = 92		*n* = 25	
	Number	Percent	Number	Percent	Number	Percent
Age						
< 45	4	2.2	3	3.3	1	4.0
45–54	30	16.3	23	25.0	1	4.0
55–64	66	35.7	37	40.2	8	32.0
65–74	53	28.8	23	25.0	9	36.0
75–84	27	14.7	6	6.5	6	24.0
> 85	4	2.2	0	0.0	0	0.0
Sex						
male	143	77.7	71	77.2	20	80.0
female	41	22.3	21	22.8	5	20.0
Diagnosis class						
first diagnosis	161	87.5	83	89.2	22	88.0
disease recurrence	23	12.5	9	9.8	3	12.0
Localization						
Oropharynx	78	42.4	40	43.5	25	100.0
Larynx	37	20.1	17	18.5	0	0.0
Hypopharynx	35	19	20	21.7	0	0.0
Oral cavity	34	18.5	15	16.3	0	0.0
Primary Tumor (T)						
cT1–2	37	20.1	13	14.1	3	12.0
cT3–4	147	79.9	79	85.9	22	88.0
Lymph Nodes (N)						
cN0-cN2a	72	39.1	35	38.0	8	32.0
cN2b-cN2c	105	57.1	53	57.6	17	68.0
cN3	7	3.8	4	4.3	0	0.0
Metastasis (M)						
cM0	184	100.0	92	100.0	25	100.0
cM1	0	0.0	0	0.0	0	0.0
Stage 7th edition (8th edition HPVOPC ^a^)						
UICC I	1	0.5	1	1.1	0 (2^a^)	0.0 (8.0 ^a^)
UICC II	8	4.3	5	5.4	1 (10 ^a^)	4.0 (40.0 ^a^)
UICC III	30	16.3	15	16.3	4 (13 ^a^)	16.0 (52.0 ^a^)
UICC IV	145	78.8	71	77.2	20 (0 ^a^)	80.0 (0.0 ^a^)
Grading						
G1	7	3.8	3	3.3	0	0
G2	89	48.8	43	46.7	9	36.0
G3	83	45.8	45	48.9	16	64.0
Gx	5	2.7	1	1.1	0	0
p16 staining						
Positive	35	19.0	21	22.8	25	100.0
Negative	121	65.8	59	64.1	0	0.0
unknown	28	15.2	12	13.0	0	0.0
smoking history						
< 10 pack-years	10	5.4	3	3.3	2	8.0
> 10 pack-years	99	53.8	49	53.3	7	28.0
unknown	75	40.8	40	43.5	16	64.0
Therapeutic Dose						
Median	70 Gy		70 Gy		70 Gy	
> = 66 Gy	179	97.3	90	97.8	25	100.0
< 66 Gy	5	2.7	2	2.2	0	0.0
GTV volume						
available	174	94.6	87	94.6	24	96.0
missing	10	5.4	5	5.4	1	4.0
range (in cc)	2–789		2–789		18–359	
mean (in cc)	61		106		107	
Chemotherapy						
no	18	9.8	0	0.0	1	4.0
yes	166	90.2	92	100.0	24	96.0
MMC / 5FU	92	50	92	100.0	18	72.0
MMC mono	28	15.2	0	0.0	2	8.0
Platin based	26	14.1	0	0.0	1	4.0
Cetuximab mono	20	10.9	0	0.0	3	12.0
Chemo completed	135	81.3	84	91.3	20	83.3
Chemo stopped	31	18.7	8	8.7	4	16.7
patient refused	1		1		0	
worsening condition	15		5		4	
cytopenia	9		1		0	
reaction to chemo	6		1		0	
Causes of death						
tumor related	50		27		1	
comorbidities	40		21		6	
therapy-associated	0		0		0	
second primary	11		5		0	
unknown	12		5		1	

^a^The UICC 8th edition stage is shown in parenthesis (HPVOPC only)

### Tumor control rates and survival data for all patients

**Fig. 1:**
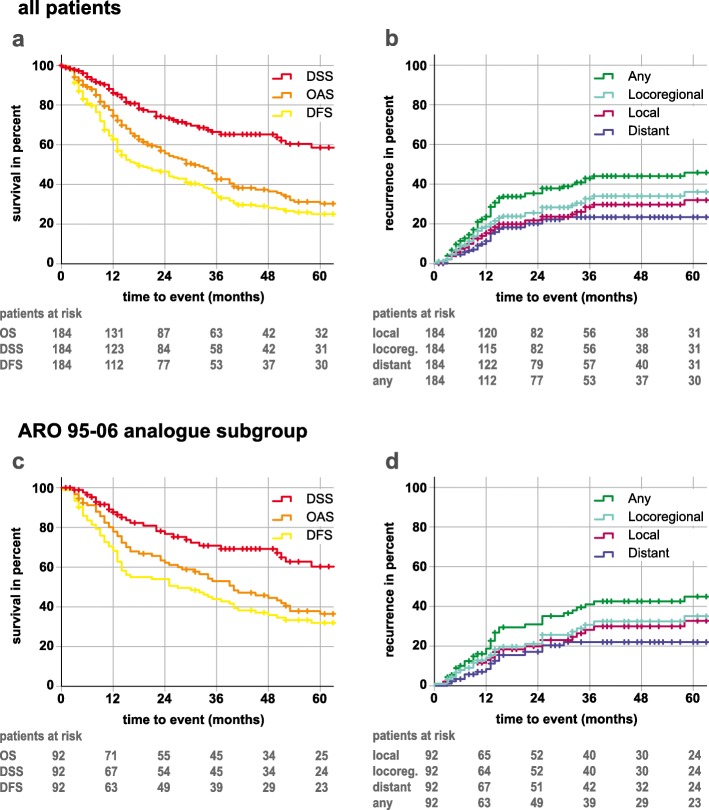
Kaplan-Meier plots **a** overall survival (OS), disease-free survival (DFS) and disease-specific survival (DSS) of all patients **b** local, locoregional, distant and any failure rates of all patients. **c** overall survival (OS), disease-free survival (DFS) and disease-specific survival (DSS) of the ARO-analogue subgroup **d** local, locoregional, distant and any failure rates of the ARO-analogue subgroup. Follow-up time was clipped at 60 months. Patients at risk are displayed under the respective plots. Censors are represented by crosses

For all patients 2-, 3- and 5-year actuarial survival rates were 55.7, 42.7 and 30.3% for overall survival (OS), 44.0, 33.8 and 24.2% for disease-free survival rates (DFS) and 73.3, 65.2 and 58.5% for disease-specific survival (DSS), respectively (Fig. 1a). The actuarial 1-, 2- and 3-year failure rates were 15.5, 23.8 and 29.8% for local, 20.0, 28.3 and 34.0% for locoregional, 15.0, 22.2 and 23.4% (last event occurred at 30 months) for distant and 23.7, 37.9 and 44.1% for all failures (Fig. 1b).

### Tumor control rates and survival data for the ARO 95–06 subgroup

Ninety-two patients were treated with MMC/5-FU in concordance to the chemotherapy arm of the ARO 95–06 study, albeit with normofractionation. The median age was 61 years (23–78 years) at time of diagnosis. The median follow-up was 70 months (see Table 1). 91% of patients received complete courses of chemotherapy; the remaining patients did not receive both cycles due to various reasons (worsening condition, refusal, cytopenia, reaction to chemotherapy). All in all, the ARO 95–06 chemotherapy regimen was well tolerated. The estimated 3-year OS, DFS and DSS were 50.6, 42.8 and 69.2%, respectively (Fig. 1c). The estimated 3 yr local, locoregional and distant failure rates were 30.0, 32.4 and 22.1%, respectively (Fig. 1d). HPV-p16-status was associated with a significantly improved locoregional control, DFS and OS in the ARO-analogue group. Compared to platinum-based chemotherapy regime no difference was found in locoregional or distant control and for DFS or OS.

### Stratification according to risk factors

**Fig. 2:**
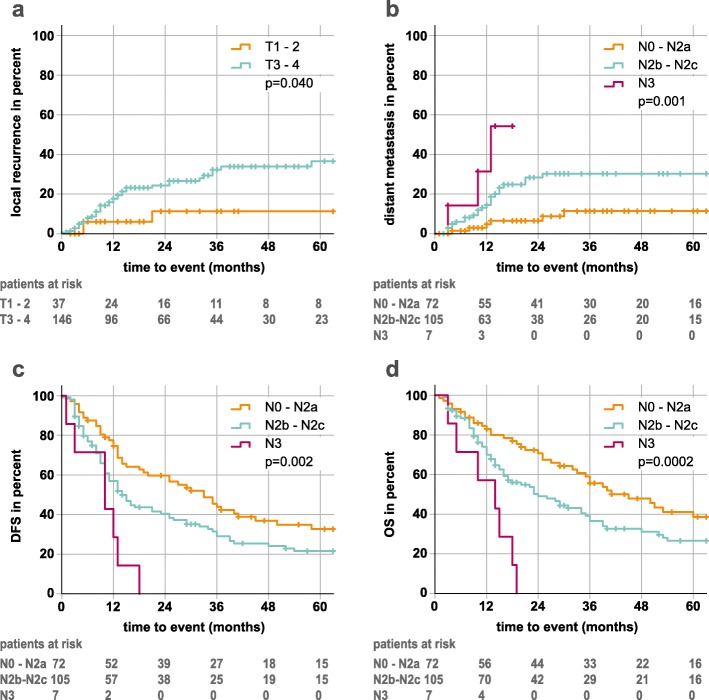
Exemplary Kaplan-Meier plots for clinical risk factors. **a** local recurrence and primary tumor size (T1–2 vs T3–4) **b** distant metastasis, **c** disease free survival, **d** overall survival and lymph node status (N0-N2a vs N2b-N2c vs N3). *P*-values (log rank) of the Kaplan-Meier estimates are shown. Follow-up time was clipped at 60 months. Patients at risk are displayed under the respective plots. Censors are represented by crosses

While the size of primary tumor (using T-stage) predicted for local recurrence only, the extent of lymph node involvement had an impact on distant metastasis rate, DFS and OS (Fig. 2). By analyzing primary tumor size using the gross tumor volume (GTVp) as continuous variable for cox regression modeling, the probability of a local relapse following dCRT increased by 4% per 10 cc absolute tumor volume. DFS and OS decreased by 3% per 10 cc in uni- and multivariate analysis (Table 2).

**Table 2:** Univariate and multivariate cox regression analysis on local, locoregional, distant and overall failure rates and overall (OS), disease-specific (DSS) and disease-free (DFS) survival rates; HPV positive oropharyngeal carcinoma (HPVOPC) and lymph node status (> = N2c) were tested as categorial variables. Gross tumor volume (GTV in cubic centimetres) and Hemoglobine (in g/dl) were tested as continuous variables

	Univariate			Multivariate		
	HR	95% CI	*p*-value	HR	95% CI	*p*-value
Local failure (LF)						
HPVOPC	0.22	0.05–0.90	0.035*	0.21	0.05–0.89	0.035*
GTV (continuous per 10 cc)	1.04	1.02–1.07	0.0004*	1.04	1.02–1.07	0.0004*
> =N2c	1.36	0.72–2.55	0.342	–	–	–
Hemoglobine (continuous per g/dl)	0.87	0.74–1.02	0.082	–	–	–
Locoregional failure (LRF)						
HPVOPC	0.18	0.44–0.76	0.019*	0.18	0.04–0.73	0.017*
GTVp (continuous per 10 cc)	1.04	1.02–1.07	0.001*	1.04	1.02–1.07	0.001*
> =N2c	1.52	0.86–2.17	0.150	–	–	–
Hemoglobine (continuous per g/dl)	0.86	0.74–1.001	0.052	–	–	–
Distant failure (DF)						
HPVOPC	1.15	0.43–3.07	0.787	–	–	–
GTV (continuous per 10 cc)	1.01	0.97–1.05	0.590	–	–	–
> =N2c	2.85	1.42–5.74	0.003*	–	–	–
Hemoglobine (continuous per g/dl)	0.95	0.79–1.14	0.549	–	–	–
Any failure (AF)						
HPVOPC	0.39	0.16–0.99	0.047*	0.37	0.15–0.94	0.037*
GTV (continuous per 10 cc)	1.04	1.01–1.06	0.001*	1.03	1.01–1.05	0.018*
> =N2c	1.95	1.20–3.18	0.007*	1.90	1.07–3.37	0.029*
Hemoglobine (continuous per g/dl)	0.89	0.77–1.02	0.090	–	–	–
Overall survival (OAS)						
HPVOPC	0.30	0.15–0.63	0.001*	0.27	0.12–0.59	0.001*
GTV (continuous per 10 cc)	1.03	1.02–1.05	0.0001*	1.03	1.01–1.04	0.010*
> =N2c	1.82	1.25–2.65	0.002*	1.48	0.96–2.27	0.073
Hemoglobine (continuous per g/dl)	0.88	0.80–0.97	0.009*	0.95	0.85–1.06	0.335
Disease specific survival (DSS)						
HPVOPC	0.09	0.01–0.63	0.016*	0.09	0.01–0.69	0.020*
GTV (continuous per 10 cc)	1.04	1.02–1.07	0.0003*	1.03	1.004–1.06	0.027*
> =N2c	2.57	1.47–4.49	0.001*	2.23	1.15–4.34	0.018*
Hemoglobine (continuous per g/dl)	0.84	0.73–0.98	0.023*	0.91	0.76–1.08	0.268
Disease free survival (DFS)						
HPVOPC	0.38	0.20–0.71	0.003*	0.37	0.19–0.71	0.003*
GTV (continuous per 10 cc)	1.03	1.02–1.05	0.00006*	1.03	1.01–1.05	0.004*
> =N2c	1.65	1.16–2.34	0.006*	1.49	0.99–2.24	0.056
Hemoglobine (continuous per g/dl)	0.90	0.82–0.97	0.024*	0.96	0.87–1.07	0.438

*P-values < 0.05 were marked with asterisk

Lower hemoglobin levels were significantly associated with impaired DFS und OS with a hazard ratio of 0.90 (*p* = 0.024) und 0.88 (*p* = 0.009) per g/dl.

With regard to the clinical endpoints there were no significant differences depending on the smoking status.

### HPV- p16 positive oropharyngeal carcinoma (HPVOPC)

**Fig. 3:**
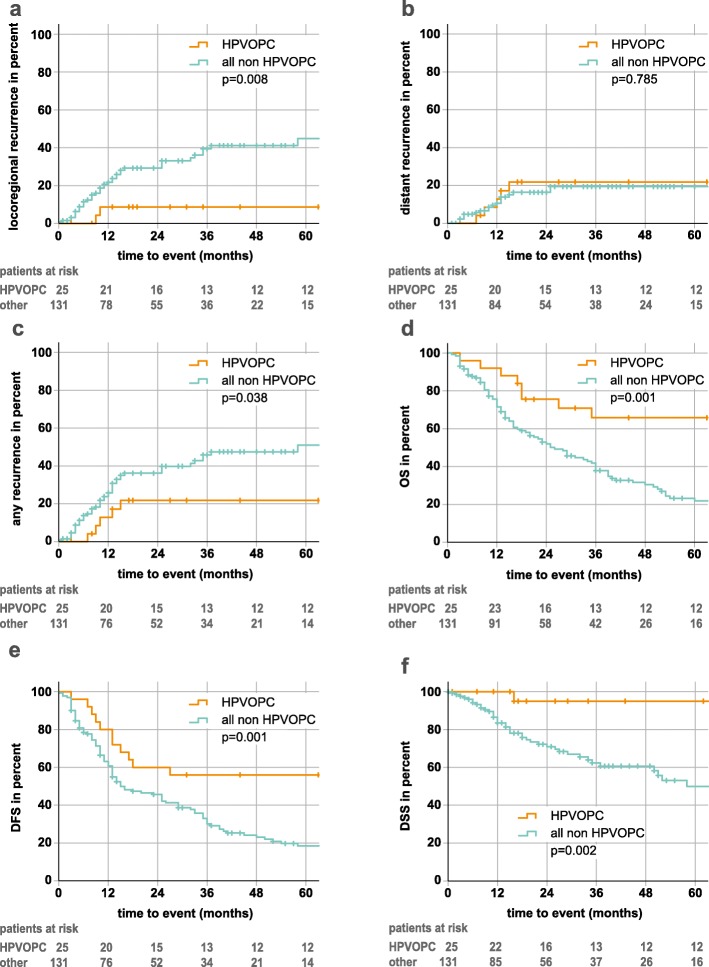
Kaplan-Meier plots for patients with HPV-p16-positive oropharyngeal cancer (HPVOPC) vs all other patients (non HPVOPC). **a** locoregional recurrence **b** distant recurrence **c** any recurrence **d** overall survival (OS) and **e** disease free survival **f** disease-specific survival (DSS). *P*-Values (log rank) of the Kaplan-Meier estimates are shown. Follow-up time was clipped at 60 months. Patients at risk are displayed under the respective plots. Censors are represented by crosses

The 3-year OS, DFS and DSS rates of HPVOPC with 65.8, 56.0 and 95.0% (last events at 35, 27 and 16 months) were significantly higher compared to 37.9, 30.2 and 60.7% of patients with non-HPVOPC, respectively (Fig. 3). Patients with HPVOPC also had significantly less local and locoregional recurrences in univariate (HR = 0.22 and 0.18, *p*-values< 0.05) and multivariate analysis (HR =0.21 and 0.18, *p*-values < 0.05). For distant failure no significant difference was found. No locoregional recurrence occurred in patients with stage I + II HPVOPC (UICC TNM 8th edition), although accounting for 48.0% of all 25 patients. Additionally, only one out of five distant failures was observed in stage I + II (8th edition) patients. For patients with HPVOPC, smoking status is known in 9 out of 25 patients only. Two of the nine patients have less than 10 pack-years and therefore meet the inclusion criteria of de-escalation studies which exclude all heavy smokers with HPVOPC. Due to the small number of cases in this subgroup, no separate analysis could be performed.

## Discussion

The present study represents a well-established and closely monitored unselected cohort of 184 “everyday patients” who were treated with definitive CRT between 09/2008 until 03/2016 in our department with tumors of the oral cavity, oropharynx, hypopharynx and larynx. Since a combined treatment by chemotherapy and radiotherapy has shown a survival benefit in many prospective studies, simultaneous CRT has become the therapeutic standard for patients with HNSCC. Depending on tumor localization the absolute survival advantage is between four to 9 % [2]. Beyond that, additional induction chemotherapy prior to concurrent CRT or acceleration of radiotherapy did not improve outcome [11–13].

The results of our patients compare favorably with previously published multicentric cohorts such as GORTEC trial [12], Head and Neck Intergroup trial [14] and ARO 95–06 trial [15].

Exemplarily, the reported 3-year overall survival was between 37 and 43% compared to 42.7% in our cohort. Our institutional chemotherapy regime for dCRT at that time was derived from the ARO 95–06 trial [16]. However, since the hyperfractionated accelerated radiation therapy used in the ARO trial in combination with chemotherapy did not have a survival advantage compared to normofractionation in other studies, we mainly treated patients with 70Gy (2 Gy per fraction, 5 fractions a week) plus mitomycin C (MMC) and 5-FU [12]. Radiotherapy with MMC/5-FU was well tolerated and fully applied in 84 out of 92 patients (91.2%). With the limitation of the small number of patients in this study and without any difference between MMC-based and platin-based chemotherapy for all endpoints, MMC/5-FU could at least be considered as an alternative therapeutic option. However, in the published literature cisplatin is reported as the standard treatment for a simultaneous monotherapy with the strongest effect [17, 18]. The clinical results of our ARO-analog subgroup and the ARO 95–06 trial were comparable with a 5-year PFS of 30.4% versus 29.3% and a 5-year OS of 36.4% versus 28.6%.

A categorical comparison of T-stage 1/2 versus 3/4 showed a significant difference with respect to local recurrences (3 years local control: 88.7% vs. 66.1%). However, this improved local control does not result in an improved DFS or OS. GTV volume, on the other hand, allowed a more detailed analysis of local tumor extension and showed a significant decrease for local and locoregional control by 4% per 10 ml tumor volume each and for DFS and OS by 3% per 10 ml tumor volume each.

Interestingly, these findings are only partially in line with a recently published paper where GTV primary tumor was only a significant independent prognostic factor for OS in p16-negative tumors but without influence on locoregional control and DFS [19].

An extended lymph node involvement (> = N2c) was associated with an increased risk for distant metastases (HR = 2.85, *p* = 0.003). This influence was also evident for OS, DFS and DSS (HR = 1.82, 1.65 and 2.57, *p*-values< 0.05) in univariate analysis, but remained significant only for DSS in multivariate analysis (HR = 2.23, *p* = 0.018). This effect may be explained by deaths from comorbidities. The results were consistent with other studies that have shown the predictive value of lymph node involvement on distant metastasis in head and neck cancer [20–22].

Additionally, the measured hemoglobin levels before radiotherapy were associated with survival. For each reduced hemoglobin unit (in g/dl) the DFS and OS decreased by a hazard ratio of 1.11 and 1.14 (*p* = 0.024 and *p* = 0.009). Anemia is common among HNSCC patients. The hemoglobin levels for 15 women and 65 men were below 12 and 13 g/dl, respectively, resulting in anemia rates of 45.5 and 36.6%. Anemic conditions before treatment may be attributed to the disease itself, impaired dietary intake and comorbid conditions of HNSCC patients [23–25]. Both in primary radiochemotherapy and in surgical approaches, the pretherapeutic haemoglobin level, the number of red blood cells and the need for blood transfusions could be identified as prognostically relevant markers for survival of head and neck cancer patients [26–28]. Unfortunately, due to the retrospective nature of this analysis, ECOG performance score was not systematically recorded, thus representing a weakness of this study.

Tumor hypoxia in HNSCC is important for predicting treatment outcomes and prognosis. There is evidence for correlations between prognosis and biomarkers with poor tumor oxygenation such as HIF-1α, GLUT-1 and lactate [29].

The use of genetic markers is increasing. Current studies use a 15-gene signature for the characterization of hypoxia [3]. In a phase III trial patients are treated with the hypoxic radiosensitizer nimorazole in addition to primary chemoradiotherapy to improve the locoregional control rate [30].

HPV-negative HNSCC and HPVOPC are two distinct clinical entities. The genesis is based on different risk factors such as years of exposure to mutagenic noxae (e.g., tobacco and alcohol) or HPV infection. The prognostic value of HPV has been confirmed in many post-hoc analyses of randomized controlled trials [4, 31–34]. This has been taken into account in the latest version of the TNM classification [35].

In this study patients with HPVOPC also had a favorable outcome compared to other HNSCC patients (OS HR = 0.27; 95% KI 0.12–0.59; *p* = 0.001 and DFS HR = 0.37; 95% KI 0.19–0.71; *p* = 0.003). The 5-year locoregional tumor control of 91.2% and the DSS of 95.0% represent the basis for discussion whether a de-escalation of the therapy is possible in order to reduce side effects without compromising the good prognosis.

In this context, different strategies could be considered: firstly, replacing cisplatin by a less toxic substance in systemic therapy; secondly, decreasing the radiation therapy dose. This could also be done in combination with induction chemotherapy to evaluate the response and differentiate between patients with good and bad prognosis.

Unfortunately, the first approach has failed so far in two recently published phase III trials [36, 37]. The De-ESCALaTE study randomly assigned HPVOPC patients to receive radiotherapy (70 Gy in 35 fractions within 7 weeks) with either cisplatin (100 mg/m2 on days 1, 22, 43) or cetuximab (400 mg/m2 loading dose followed by 250 mg/m2 weekly). Acute and late toxicity did not differ significantly between treatment groups at 24 months. However, a significant difference between cisplatin and cetuximab in 2-year overall survival (97.5% vs 89.4%) and 2-year any recurrence (6.0% vs 16.1%) was seen [36]. The RTOG1016 had the same treatment approach except for the acceleration of radiotherapy (70 Gy in 35 fractions within 6 weeks). Proportions of acute and late moderate to severe toxicity were similar between the cetuximab and cisplatin groups. Estimated 5-year overall survival was significantly lower and locoregional failure significantly higher in the cetuximab group compared to the cisplatin group (5-years OS 77.9% vs 84.6%; 5-years LRF 17.3% vs 9.9%) [37]. Another phase III randomized trial (TROG 12.01) treating patients with radiotherapy (70Gy in 35 fractions within 7 weeks) and cisplatin (40 mg/m^2^ weekly) or cetuximab is still ongoing.

For the second approach (reduction of radiation dose) there are a number of heterogeneous studies with partly promising results. In a phase III trial 200 patients were randomly assigned to either receive 50Gy or 40Gy only to the elective radiation volumes [38]. The trial included all HNSCC irrespective of HPV status. The primary endpoint was dysphagia. In the 40 Gy group a trend was observed toward less dysphagia at 6 months and less moderate salivary gland toxicity without significant differences in disease control (locoregional failure rates 24% vs 15%, *p* = 0.14) or survival (OS 72 and 73% *p* = 0.73). However, the results for disease control should be considered with caution as this was not a non-inferiority analysis with a sufficient number of patients.

Several other trials used a combination of induction chemotherapy and radiation dose reduction. The favorable results showed survival rates above 90%. In addition to the clinical and radiological interim evaluation of the tumor’s therapeutic response as a surrogate for biological aggressiveness and resistance to cytotoxic therapies, induction chemotherapy in theory also offers the possibility of eliminating distant micrometastases. In our cohort 21.8% of HPVOPC patients had distant metastasis at 2 years. Due to salvage options this did not influence disease specific survival.

The OPTIMA phase II trial stratified patients into a low risk and a high risk group depending on tumor size and lymph node involvement [39]. After 3 cycles of carboplatin and Nab-paclitaxel, the patients were assigned to three treatment arms depending on the radiological assessment of the response. At radiological response rates < 30%, 30–50%, or > 50%, low-risk patients received 45 Gy, 30 Gy or no radiotherapy on elective volume and 75 Gy, 75 Gy or 50 Gy on macroscopic tumor. At a response rate of < 50% or > 50%, high-risk patients were treated with 45 Gy or 30 Gy in elective volume and generally 75 Gy on macroscopic tumor. The 2-years OS and PFS were both 100% for low risk and 97.0 and 92.2% for high risk group. In another phase II trial (ECOG 1308) using induction chemotherapy (3 cycles of cisplatin, paclitaxel, cetuximab) followed by reduced-dose radiation (54 Gy in 26 fractions) and weekly cetuximab clinical responders with low risk features (non-T4, non-N2c, <10PY) had a 2-years PFS and OS of 96 and 96% [40].

The phase III Quarterback Trial comparing standard (70 Gy) versus low dose (56 Gy) with weekly cetuximab plus carboplatin or carboplatin only, depending on the response to induction chemotherapy (3 cycles of TPF) is still ongoing.

Outside of clinical trials, a de-escalation of the therapy of HPVOPC cannot be recommended. At present, platin-based fully dosed dCRT remains the treatment standard. Compared to the postoperative cohort (surgery and adjuvant chemoradiotherapy) of our clinic, dCRT alone resulted in comparable locoregional tumor control rates for HPVOPC (3-year locoregional failure 4.6% vs 8.7%) [41]. A resection of locoregionally advanced HPVOPC with the consequence of significant functional impairment should remain the exception due to the excellent results of dCRT [42].

## Conclusion

Overall, the presented monocentric cohort containing “everyday patients” treated with dCRT, confirms the known risk factors previously described with robust clinical data. Thus, it is in line with the results of published cohorts. Further translational research based on this dCRT HNSCC cohort is already ongoing within the framework of the clinical cooperation group “Personalized Radiotherapy for Head and Neck Cancer”.

## Data Availability

The datasets generated and/or analyzed during the current study are not publicly available due to privacy regulations in the ethics approval but are available from the corresponding author on reasonable request.
